# Predictive performance of Triple-D, Quadruple-D, and Mayo adhesive probability scores in ESWL for renal stones: a retrospective cohort study

**DOI:** 10.1007/s00240-025-01765-6

**Published:** 2025-05-22

**Authors:** Osman Murat Ipek, Erdinc Dincer, Ahmet Halil Sevinc, Burcu Hanci Sevinc, Cengiz Canakci, Orkunt Ozkaptan

**Affiliations:** Department of Urology, Kartal Dr. Lutfi Kirdar City Hospital, Cevizli Mevkii No:47, D-100, Kartal, İstanbul, 34865 Türkiye

**Keywords:** Urolithiasis, Extracorporeal shockwave lithotripsy, Treatment outcome, Predictive value of tests, Scoring systems

## Abstract

This study aimed to compare the predictive performance of Triple-D, Quadruple-D, and Mayo Adhesive Probability (MAP) scoring systems in estimating stone-free (SF) status following extracorporeal shock wave lithotripsy (ESWL) in patients with renal stones. This retrospective cohort study was conducted on patients who underwent ESWL between January 2020 and January 2024. Pre-treatment non-contrast computed tomography was used to assess stone characteristics and calculate Triple-D, Quadruple-D, and MAP scores. Patients were categorized into stone-free (SF) and residual stone (RS) groups based on imaging performed three months after treatment. Residual fragments of < 4 mm were defined as SF. The study included 198 patients (60.6% male; mean age 45.5 ± 13.1 years). According to logistic regression analysis, a low MAP score (< 2.5) was the strongest independent predictor of SF status (OR: 15.5; 95% CI: 5.1–47.1; *p* < 0.001), followed by a high Quadruple-D score (> 1.5) (OR: 7.4; 95% CI: 2.2–24.1; *p* = 0.001) and low stone density (< 600 HU) (OR: 4.9; 95% CI: 1.1–21.8; *p* = 0.037). Conversely, a higher number of shockwaves and the need for additional procedures were associated with RS (both *p* < 0.001). Among the scoring systems, MAP score demonstrated the highest predictive accuracy with an AUC of 0.817, outperforming Quadruple-D (AUC: 0.722) and Triple-D (AUC: 0.639). MAP score was the most powerful and accurate independent predictor of SF status after ESWL, offering superior clinical utility compared to Triple-D and Quadruple-D scores in pre-treatment evaluation.

## Introduction

Renal stones have always been one of the most common urological conditions and global estimates show an increasing incidence due to dietary and environmental factors [1]. While many stones pass spontaneously, a significant proportion require active interventions due to obstruction or infection [2]. Extracorporeal shock wave lithotripsy (ESWL) is the well-established first-line therapeutic option for many types of renal stones, particularly those smaller than 20 mm in diameter [3]. It can be administered in an outpatient setting, has low complication rates, and is cost-effective; however, curative treatment (stone-free; SF) frequencies vary significantly based on patient and stone characteristics, as well as other features [4].

Numerous studies have attempted to identify factors influencing ESWL outcomes, with stone size, volume, density, location, skin-to-stone distance (SSD), and body mass index frequently reported as significant predictors [4–8]. To incorporate these radiological parameters into clinical decision-making, composite scoring systems such as the Triple-D score (dimension, density, and SSD) and the Quadruple-D score (adds stone location) have been developed [9]. These models aim to simplify and standardize pre-treatment assessment, but they are known to have limitations. The Mayo Adhesion Probability (MAP) score was originally designed to estimate surgical difficulty in percutaneous nephrolithotomy. More recently, MAP score has shown potential utility in predicting ESWL outcomes by evaluating perinephric fat stranding and thickness [10]. However, direct comparisons between these scoring systems in predicting ESWL success remain limited in the literature.

Our purpose was to evaluate and compare the predictive performances of the Triple-D, Quadruple-D, and MAP scoring systems in determining SF status after ESWL in patients with renal stones. By comparing the relatively recent MAP to previously described predictors, this study seeks to contribute evidence that can improve pre-treatment assessment in patients undergoing ESWL.

## Materials and methods

### Study design and ethical approval

This retrospective cohort study was conducted at the Urology Department of Kartal Dr. Lütfi Kırdar City Hospital between January 2020 and January 2024. The clinical and radiological data of adult patients (≥ 18 years) who underwent ESWL for renal stones were analyzed. The study was conducted in accordance with the principles of the Declaration of Helsinki and was approved by the Institutional Clinical Research Ethics Committee of Kartal Dr. Lütfi Kırdar City Hospital (Decision date: 24.01.2025, decision no: 2025/010.99/12/27).

### Patient selection

Patients were included if they were ≥ 18 years old, had undergone ESWL for renal stones, and their follow-up data were complete. In addition, we only included individuals who had undergone abdominal computed tomography (CT) imaging before treatment. Patients were excluded if they lacked pre-procedural CT imaging, did not complete the ESWL sessions, had anatomical abnormalities of the urinary tract (such as ureteropelvic junction obstruction, horseshoe kidney, or duplex systems), had renal pathology unrelated to urolithiasis, or had incomplete clinical or radiological data.

### Clinical data and parameters

Demographic data including age, sex, body mass index, stone laterality (right/left), fluoroscopy time (seconds), number of ESWL sessions, total number of shockwaves delivered, post-procedural complications (e.g., pyelonephritis, steinstrasse), and any additional interventions (double-J stent placement, nephrostomy, ureteroscopy, or retrograde intrarenal surgery) were retrieved from the hospital records.

### ESWL protocol

All ESWL procedures were performed using a Dornier Compact Sigma (Med Tech, Munich, Germany) lithotripter, by three urologists who were similarly experienced in the routine ESWL procedure. Targeting was achieved using a combination of fluoroscopy and ultrasonography. Prior to the procedure, urine cultures were obtained from all patients and sterile status was confirmed. If necessary, appropriate antibiotic therapy was initiated before beginning treatment for renal stones. ESWL was initiated at a rate of 60 shocks per minute and gradually increased to 90 shocks/min depending on patient tolerance [11]. A maximum of four sessions were allowed per patient, with at least one week between sessions.

### Evaluation of treatment outcome

Treatment success was evaluated three months after completion of ESWL sessions using follow-up imaging, including non-contrast abdominal CT, urinary tract ultrasonography, and kidney-ureter-bladder radiography. Patients with residual stone (RS) size of > 4 mm were not considered to have received curative treatment and were defined as having residual stones. However, fragments of < 4 mm were considered sufficient to proclaim SF status based on current literature supporting spontaneous passage of fragments below this threshold [12]. Complications occurring within 30 days of treatment and any additional procedures were also recorded.

### Radiological evaluation

The pre-treatment CT imaging studies performed in all patients were routinely non-contrasted spiral CTs (Siemens Somatom Definition AS, Germany; 1-mm slice thickness). Radiological analysis was performed using high-resolution axial and coronal sections on the institutional PACS system. Stone dimensions were measured in three axes (X: length, Y: width, Z: depth), and stone volume was calculated using the ellipsoid formula: X × Y × Z × π / 6. Stone density (HU) was determined by averaging three measurements taken from the center of the stone. SSD was measured at a 45-degree angle from the center of the stone to the skin in the axial plane. Stone location was categorized as upper calyx, middle calyx, lower calyx, renal pelvis, or ureteropelvic junction. Hydronephrosis was graded radiologically from Grade 0 (none) to Grade 4 (severe) [13].

### Scoring systems

Three scoring systems were calculated for each patient using CT parameters: Triple-D, Quadruple-D, and MAP scores. All radiological measurements and scoring assessments were performed by a single radiologist specializing in uroradiology who was blinded to clinical outcomes [14].

The Triple-D score assigned one point each for stone volume < 150 mm³, stone density < 600 HU, and SSD < 12 cm, resulting in a total score between 0 and 3 [15].

The Quadruple-D score included the three parameters of Triple-D and added one point for stones located in the lower calyx, with a total score range of 0 to 4 [16].

The MAP score was calculated based on the thickness of perinephric fat at the level of the renal hilum and the degree of perinephric fat stranding. Stranding was graded as 0 (none), 1 (mild), or 2 (marked). Perinephric fat thickness was scored based on a dichotomization value (≥ 10 mm), with values higher than this threshold receiving 1 point, while lower values received 0 points. As such, total MAP score ranged from 0 to 5.

### Statistical analysis

All analyses were conducted using IBM SPSS version 25.0 (IBM Corp., Armonk, NY, USA). Two-tailed p-values of less than 0.05 were accepted as statistically significant. The Kolmogorov-Smirnov test was used to exclude normal distribution. Descriptive statistics for continuous variables were presented using mean ± standard deviation or median (25th percentile − 75th percentile) depending on normal or non-normal distribution, while frequency (percentage) was used for categorical variables. Between groups analysis of continuous variables were performed using the Student’s t test or Mann Whitney U test depending normality of distribution. Categorical analyses were performed using chi-square tests or the Fisher-Freeman-Halton test. Performance of scores to predict SF status were evaluated using the receiver operating characteristic (ROC) curve analysis (cut-offs identified with Youden’s index). Multivariable logistic regression (forward conditional selection) was performed to determine factors independently associated with SF status.

## Results

A total of 198 patients were included in the study, of whom 60.6% were male. The mean age was 45.52 ± 13.10 years (range: 20–82 years). When compared to the RS group, the SF group demonstrated significantly shorter fluoroscopy duration and smaller stone volume, whereas stone density was significantly lower (*p* < 0.001 for all). In the RS group, stones were more commonly located in the lower calyx (40.9%) and renal pelvis (34.9%), while in the SF group, the majority of stones (65.0%) were located in the renal pelvis (*p* < 0.001). The SF group also had significantly lower hydronephrosis grade (*p* = 0.016), fewer treatment sessions (*p* < 0.001), fewer shockwaves delivered (*p* < 0.001), lower rates of additional procedures (*p* < 0.001), and fewer complications (*p* < 0.001) compared to those with RSs. In terms of scoring systems, the SF group had significantly higher Triple-D and Quadruple-D scores, and a significantly lower MAP score (*p* < 0.001 for all) (Table 1).

**Table 1:** Summary of demographics, stone and intervention characteristics, and scores with regard to stone-free status

Variables	Total (*n* = 203)	Stone-free (*n* = 137)	Residual stone (*n* = 66)	*p*
Age	45.52 ± 13.10	44.88 ± 13.13	46.85 ± 13.02	0.316^†^
Sex				
Male	123 (60.59%)	79 (57.66%)	44 (66.67%)	0.282^§^
Female	80 (39.41%)	58 (42.34%)	22 (33.33%)	
Body mass index, kg/m^2^	27.45 ± 4.19	27.59 ± 4.19	27.18 ± 4.21	0.514^†^
Side				
Right	110 (54.19%)	69 (50.36%)	41 (62.12%)	0.115^§^
Left	93 (45.81%)	68 (49.64%)	25 (37.88%)	
Duration of fluoroscopy	145 (110–210)	133 (101–195)	156 (127–258)	**< 0.001** ^**‡**^
Stone size, mm				
X (length)	12 (9.4–14)	11 (9–13.5)	13.25 (12–16)	**< 0.001** ^**‡**^
Y (width)	8 (6.6–10)	7.7 (6.3–10)	9.1 (7–11.4)	**0.003** ^**‡**^
Z (depth)	8.5 (7–11)	8 (6–10)	10.2 (8–12.1)	**< 0.001** ^**‡**^
Stone volume, mm^3^	439.82 (230.91–721.31)	368.61 (201.06–613.11)	618.87 (374.05–926.14)	**< 0.001** ^**‡**^
< 150 mm^3^	21 (10.34%)	17 (12.41%)	4 (6.06%)	0.252^§^
Stone density, HU	790 (603–1013)	715 (570–887)	998 (840–1117)	**< 0.001** ^**‡**^
< 600 HU	48 (23.65%)	43 (31.39%)	5 (7.58%)	**< 0.001** ^**§**^
Location				
Upper calyx	18 (8.87%)	17 (12.41%)	1 (1.52%)*	**< 0.001** ^**§**^
Middle calyx	21 (10.34%)	16 (11.68%)	5 (7.58%)	
Lower calyx	37 (18.23%)	10 (7.30%)	27 (40.91%)*	
Renal pelvis	112 (55.17%)	89 (64.96%)	23 (34.85%)*	
Ureteropelvic junction	15 (7.39%)	5 (3.65%)	10 (15.15%)*	
Skin-stone distance, cm	10.14 ± 1.77	10.16 ± 1.60	10.09 ± 2.09	0.808^†^
< 12 cm	169 (83.25%)	117 (85.40%)	52 (78.79%)	0.326^§^
Hydronephrosis grade				
Grade 0	28 (13.79%)	21 (15.33%)	7 (10.61%)	**0.016** ^**¶**^
Grade 1	90 (44.33%)	65 (47.45%)	25 (37.88%)	
Grade 2	68 (33.50%)	45 (32.85%)	23 (34.85%)	
Grade 3	13 (6.40%)	6 (4.38%)	7 (10.61%)	
Grade 4	4 (1.97%)	0 (0.00%)	4 (6.06%)*	
Number of sessions	2 (1–3)	1 (1–3)	3 (2–3)	**< 0.001** ^**‡**^
One	81 (39.90%)	72 (52.55%)	9 (13.64%)	
Two	45 (22.17%)	27 (19.71%)	18 (27.27%)	
Three	74 (36.45%)	38 (27.74%)	36 (54.55%)	
Four	3 (1.48%)	0 (0.00%)	3 (4.55%)	
Number of shockwaves	6000 (3000–8400)	3000 (3000–6800)	8400 (5800–8600)	**< 0.001** ^**‡**^
Additional procedure	25 (12.32%)	2 (1.46%)	23 (34.85%)	**< 0.001** ^**§**^
DJ stent insertion	7 (3.45%)	0 (0.00%)	7 (10.61%)	
Nephrostomy	5 (2.46%)	0 (0.00%)	5 (7.58%)	
URS/RIRS	13 (6.40%)	2 (1.46%)	11 (16.67%)	
Complication	23 (11.33%)	2 (1.46%)	21 (31.82%)	**< 0.001** ^**§**^
Pyelonephritis	7 (3.45%)	0 (0.00%)	7 (10.61%)	
Steinstrasse	16 (7.88%)	2 (1.46%)	14 (21.21%)	
Triple-D score	1 (1–2)	1 (1–2)	1 (1–1)	**< 0.001** ^**‡**^
Score 0	23 (11.33%)	11 (8.03%)	12 (18.18%)	
Score 1	128 (63.05%)	81 (59.12%)	47 (71.21%)	
Score 2	46 (22.66%)	39 (28.47%)	7 (10.61%)	
Score 3	6 (2.96%)	6 (4.38%)	0 (0.00%)	
Quadruple-D score	2 (2–2)	2 (2–3)	2 (1–2)	**< 0.001** ^**‡**^
Score 0	9 (4.43%)	2 (1.46%)	7 (10.61%)	
Score 1	38 (18.72%)	14 (10.22%)	24 (36.36%)	
Score 2	108 (53.20%)	79 (57.66%)	29 (43.94%)	
Score 3	42 (20.69%)	36 (26.28%)	6 (9.09%)	
Score 4	6 (2.96%)	6 (4.38%)	0 (0.00%)	
MAP score	2 (2–3)	2 (1–3)	4 (3–4)	**< 0.001** ^**‡**^
Score 0	8 (3.94%)	7 (5.11%)	1 (1.52%)	
Score 1	41 (20.20%)	36 (26.28%)	5 (7.58%)	
Score 2	63 (31.03%)	57 (41.61%)	6 (9.09%)	
Score 3	49 (24.14%)	29 (21.17%)	20 (30.30%)	
Score 4	32 (15.76%)	7 (5.11%)	25 (37.88%)	
Score 5	10 (4.93%)	1 (0.73%)	9 (13.64%)	

Descriptive statistics are presented using mean ± standard deviation for normally distributed continuous variables, median (25th percentile − 75th percentile) for non-normally distributed continuous variables and frequency (percentage) for categorical variables

Abbreviations: DJ: Double-J, HU: Hounsfield unit, MAP: Mayo adhesion probability, RIRS: Retrograde intrarenal surgery, URS: Ureterorenoscopy

† Student’s t test, ‡ Mann Whitney U test, § Chi-square test, ¶ Fisher-Freeman-Halton test, * Statistically significant category for the variables with three or more categories. Statistically significant p values are shown in bold

### Predictive performance of scoring systems

**Fig. 1:**
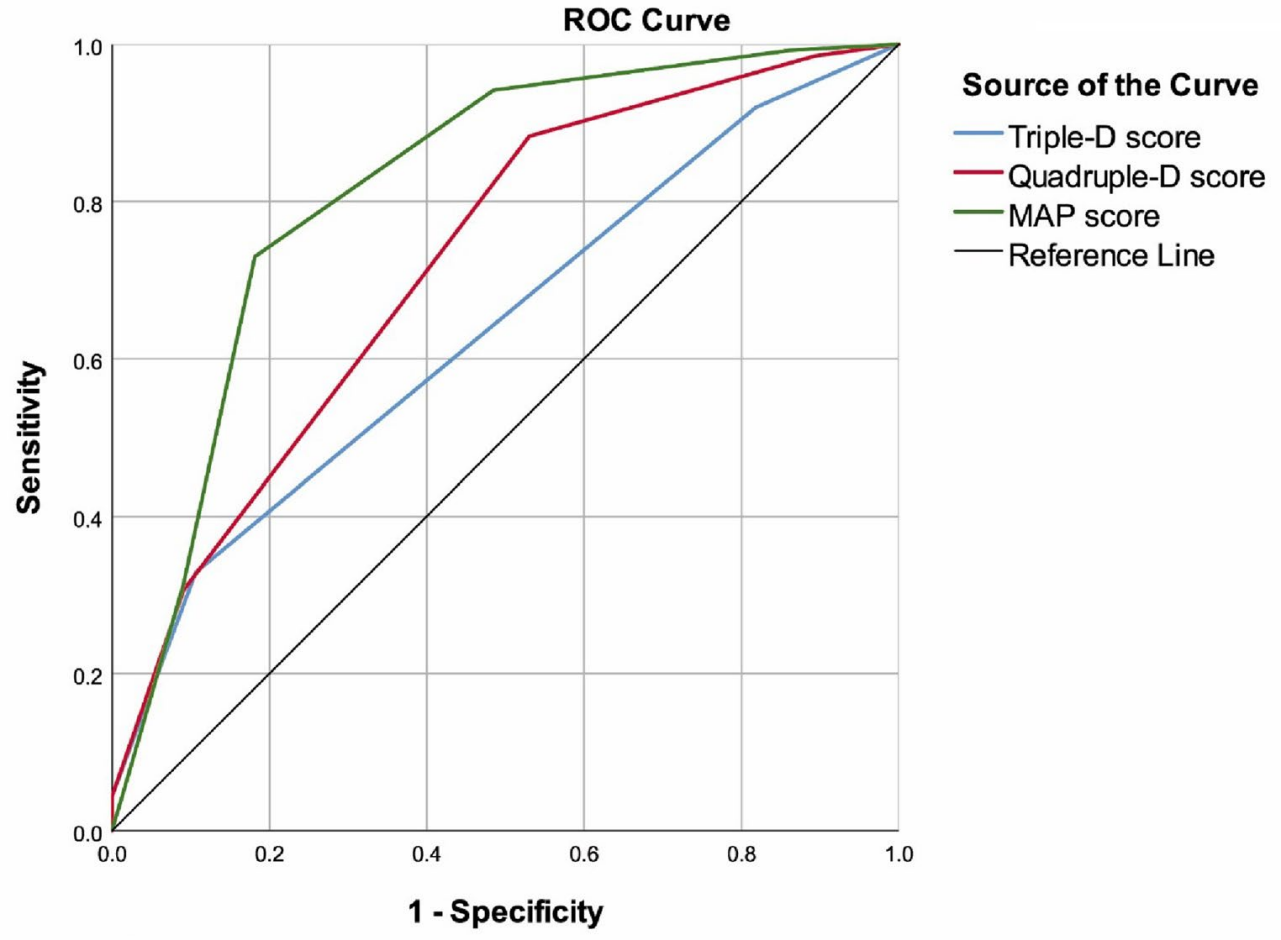
ROC curves of the scores to predict stone-free status

ROC curve analysis revealed that the MAP score had the highest discriminative ability in predicting SF status (area under curve–AUC = 0.817; 95% CI: 0.749–0.885, *p* < 0.001). Using a cut-off value of < 2.5, the MAP score yielded a sensitivity of 72.99%, specificity of 81.82%, accuracy of 75.86%, positive predictive value (PPV) of 89.29%, and negative predictive value (NPV) of 59.34%. The Quadruple-D score, with a cut-off value of > 1.5, showed a sensitivity of 88.32%, specificity of 46.97%, accuracy of 74.88%, PPV of 77.56%, and NPV of 65.96%, with an AUC of 0.722 (95% CI: 0.647–0.798, *p* < 0.001). Although the Triple-D score was more specific (89.39%), it demonstrated lower sensitivity (32.85%) and accuracy (51.23%), with an AUC of 0.639 (95% CI: 0.560–0.717, *p* = 0.001) (Table 2; Fig. 1).

**Table 2:** Performance of scores to predict stone-free outcome, ROC curve analysis

	Triple-D score	Quadruple-D score	MAP score
Cut-off	> 1.5	> 1.5	< 2.5
Sensitivity	32.85%	88.32%	72.99%
Specificity	89.39%	46.97%	81.82%
Accuracy	51.23%	74.88%	75.86%
PPV	86.54%	77.56%	89.29%
NPV	39.07%	65.96%	59.34%
AUC (95% CI)	0.639 (0.560–0.717)	0.722 (0.647–0.798)	0.817 (0.749–0.885)
p	**0.001**	**< 0.001**	**< 0.001**

AUC: Area under ROC curve, CI: Confidence interval, MAP: Mayo adhesion probability, NPV: Negative predictive value, PPV: Positive predictive value, ROC: Receiver operating characteristic. Statistically significant p values are shown in bold

### Factors independently associated with stone-free status

According to multivariable logistic regression analysis results, low (< 600 HU) stone density (odds ratio [OR]: 4.895; 95% confidence interval [CI]: 1.097–21.840; *p* = 0.037), high (> 1.5) Quadruple-D score (OR: 7.425; 95% CI: 2.285–24.128; *p* = 0.001) and low (< 2.5) MAP score (OR: 15.529; 95% CI: 5.122–47.080; *p* < 0.001) were independently associated with SF status. On the other hand, high number of shockwaves (OR: 0.999; 95% CI: 0.999–1.000; *p* < 0.001) and additional procedure need (OR: 0.010; 95% CI: 0.001–0.086; *p* < 0.001) were independently associated with RS presence. Other variables included in the analysis, duration of fluoroscopy (*p* = 0.077), stone length (*p* = 0.236), stone width (*p* = 0.849), stone depth (*p* = 0.249), stone volume (*p* = 0.320), stone location (*p* = 0.052), hydronephrosis grade (*p* = 0.160), number of sessions (*p* = 0.610), complication (*p* = 0.910) and Triple-D score (*p* = 0.103) were found to be non-significant (Table 3).

**Table 3:** Significant factors independently associated with stone-free status, multivariable logistic regression analysis

	β coefficient	Standard error	*p*	Exp(β)	95% CI for Exp(β)	
Stone density, < 600 HU	1.588	0.763	**0.037**	4.895	1.097	21.840
Number of shockwaves	-0.001	0.000	**< 0.001**	0.999	0.999	1.000
Additional procedure	-4.631	1.108	**< 0.001**	0.010	0.001	0.086
Quadruple-D score, > 1.5	2.005	0.601	**0.001**	7.425	2.285	24.128
MAP score, < 2.5	2.743	0.566	**< 0.001**	15.529	5.122	47.080
Constant	2.557	0.921	0.006	12.899		

Nagelkerke R^2^ = 0.728, CI: Confidence interval, HU: Hounsfield unit. Statistically significant p values are shown in bold

## Discussion

In this retrospective cohort study, we evaluated the predictive performance of three radiological scoring systems (Triple-D, Quadruple-D, and MAP) in estimating SF status following ESWL in patients with renal stones. The MAP score demonstrated the highest discriminative ability, with particularly improved specificity and PPV values. Multivariable analysis revealed that a low MAP score, high Quadruple-D score, and lower stone density were independently associated with achieving SF status. Conversely, higher shockwave counts and the need for additional procedures were linked to treatment failure. While all three scoring systems provided clinically relevant stratification, the MAP score had the strongest independent association with treatment outcome. These findings suggest that combining traditional stone parameters with MAP score may improve the ability to predict ESWL success and guide clinical decision-making.

The predictive roles of the Triple-D and Quadruple-D scoring systems in estimating ESWL success have been evaluated in several previous studies, and our findings are largely consistent with this existing body of literature [15, 17–20]. Both scores were significantly associated with SF status in univariate evaluation. The Quadruple-D score could be expected to improve upon the Triple-D model, since particularly lower calyceal stones are known to reduce the likelihood of ESWL success [16, 21]. Indeed, the Quadruple-D score appears to demonstrate superior predictive ability compared to the Triple-D score [9, 21, 22]. Coskun and Can reported that a cut-off value of 1.5 for the Triple-D score and 2.5 for the Quadruple-D score could serve as optimal thresholds for predicting SF status [23]. Similarly, in our study, although both scoring systems showed significant associations with SF outcomes, the observed sensitivity and specificity values indicate that these scores are not strong discriminators.

The MAP score is a radiological scoring system that incorporates perinephric fat thickness and the degree of perinephric fat stranding. In our study, it demonstrated the highest predictive accuracy among the three evaluated models. A MAP score below 2.5 was found to be strongly and independently associated with SF status, surpassing both the Triple-D and Quadruple-D scores in terms of discriminative performance. To the best of our knowledge, only one prior study in the literature has specifically examined the association between the MAP score and ESWL outcomes. In that study, conducted by Caglar et al., a MAP cut-off value of 2 was found to predict treatment success with a sensitivity of 76.5% and specificity of 64.3%, and the MAP score was identified as an independent predictor of ESWL success [10]. In contrast, a study by Haberal et al. evaluated the MAP score in patients undergoing percutaneous nephrolithotomy (PCNL), dividing patients into low (0–2) and high (3–5) MAP score groups, and reported no association with SF status [24]. Interestingly, other studies investigating PCNL outcomes have shown that higher MAP scores are associated with increased risk of postoperative fever [25, 26], intraoperative bleeding [26, 27] and other complications [28]. Our findings suggest that perirenal anatomical structures, as reflected by the MAP score, may influence clinical outcomes, particularly in the context of ESWL and stone fragmentation. Incorporating the MAP score into pre-treatment assessment alongside classical parameters such as stone density and location may provide a more comprehensive and individualized approach to predict ESWL outcomes. The limited research on this topic restricts the ability to draw more definitive conclusions, which warrants prospective and multicenter research to clarify whether MAP can replace the use of the Triple-D and Quadruple-D scores.

Rather than relying solely on traditional factors such as stone size or location, the use of composite models allows for a more accurate prediction of treatment success [23]. In particular, the MAP score may assist in identifying patients who are less likely to benefit from ESWL and for whom alternative treatment options, such as ureteroscopy or percutaneous nephrolithotomy, may be more appropriate. An additional advantage of these scoring systems is their practical applicability, as all necessary parameters can be derived from standard non-contrast CT scans routinely obtained during pre-treatment evaluation. This makes MAP a cost-effective, noninvasive, and easy-to-use tool that can support clinicians in determining management strategies based on individual characteristics.

This study has several limitations that should be taken into account. First, the retrospective design limits control over confounding variables and introduces potential bias related to data completeness and accuracy. As the data were collected from a single institution and involved a relatively limited number of patients, the findings may not be generalizable to other populations or healthcare settings. Although all ESWL sessions were performed by experienced urologists using a consistent protocol, subtle differences in individual technique, patient selection criteria, or procedural adjustments might have influenced outcomes. In addition, the MAP score, which incorporates radiological evaluation of perinephric fat characteristics, is partly based on subjective image interpretation, potentially leading to inter-observer variability. This particular limitation can be addressed with the creation of guidelines for MAP use in the assessment of such patients. Finally, we did not enroll a validation cohort and did not assess alternative treatment modalities, such as ureteroscopy or percutaneous nephrolithotomy, which could be important to assess MAP utility in patients receiving other treatments for renal stones.

Our findings demonstrated that while all three scores provided clinically relevant information, the MAP score showed the highest discriminative performance in predicting treatment success. Notably, multivariable analysis showed that a low MAP score (< 2.5), a high Quadruple-D score (> 1.5), and lower stone density were independently associated with SF status. These results suggest that incorporating comprehensive radiological parameters, including perinephric fat characteristics, may enhance the accuracy of pre-treatment assessment in ESWL candidates. Future prospective studies with larger, multicenter cohorts that include other treatment modalities are warranted to validate these findings and to explore the integration of such scoring systems into the routine clinical practice.

## Data Availability

No datasets were generated or analysed during the current study.
